# Probing the underlying principles of dynamics in piano performances using a modelling approach

**DOI:** 10.3389/fpsyg.2023.1269715

**Published:** 2023-12-12

**Authors:** Gabriel Jones, Anders Friberg

**Affiliations:** ^1^University of Leeds, School of Music, Leeds, United Kingdom; ^2^KTH Royal Institute of Technology, Stockholm, Sweden

**Keywords:** dynamics, music analysis, melody, modelling, machine learning, piano performance

## Abstract

Variations in dynamics are an essential component of musical performance in most instruments. To study the factors that contribute to dynamic variations, we used a model approaching, allowing for determination of the individual contribution of different musical features. Thirty monophonic melodies from 3 stylistic eras with all expressive markings removed were performed by 20 pianists on a Disklavier piano. The results indicated a relatively high agreement among the pianists (Cronbach’s alpha = 0.88). The overall average dynamics (across pianists) could be predicted quite well using support vector regression (R^2^ = 66%) from a set of 48 score-related features. The highest contribution was from pitch-related features (37.3%), followed by phrasing (12.3%), timing (2.8%), and meter (0.7%). The highest single contribution was from the high-loud principle, whereby higher notes were played louder, as corroborated by the written feedback of many of the pianists. There were also differences between the styles. The highest contribution from phrasing, for example, was obtained from the Romantic examples, while the highest contribution from meter came from the Baroque examples. An analysis of each individual pianist revealed some fundamental differences in approach to the performance of dynamics. All participants were undergraduate-standard pianists or above; however, varied levels of consistency and predictability highlighted challenges in acquiring a reliable group in terms of expertise and preparation, as well as certain pianistic challenges posed by the task. Nevertheless, the method proved useful in disentangling some underlying principles of musical performance and their relation to structural features of the score, with the potential for productive adaptation to a wider range of expressive and instrumental contexts.

## Introduction

This paper explores the expressive use of dynamics in piano performances of melodies in different musical styles. Dynamics have been a central and elusive focus of music research since the early experiments of Seashore (1938). Their manifestation depends on many different factors, including the style and period of the music being played (Friberg and Bisesi, 2014); the expressive sensibility (Jones, 2022), musical intentions (Gabrielsson, 1995), and biomechanical limitations (Parlitz et al., 1998) of the performer; the acoustics of the performance space (Kalkandjiev and Weinzierl, 2015); and the material characteristics of different musical instruments (Luce, 1975).

Three methodological strands have emerged in investigations of dynamics in musical performances: (1) experimental investigations of audience perception and/or aspects of human performance; (2) performance-analytical studies of existing recordings; (3) computer-assisted modelling.

Geringer and Breen (1975) surveyed experimental methods that followed Seashore (for example Fay, 1947; Gordon, 1960; Farnsworth, 1969), prior to the emergence of musical performance studies as a recognised discipline in the early 1980s (see Gabrielsson, 1999 and Gabrielsson, 2003 for comprehensive overviews of the field at the turn of the millennium). Geringer and Breen’s own experiment investigated audience perception of expression in Classical music and rock-and-roll music when performed with varying dynamic levels. They found that large dynamic changes do not enhance musical expression in the latter, and that dynamic changes in classical music are perceived to be more expressive when aligned with certain structurally significant features. While relatively simplistic, their methods of neutral versus augmented dynamic presentation, and focus on the relationship of dynamic markings, expressive realisation, and audience perception, anticipate much later research.

More detailed studies and comparisons of performed dynamics in experimental settings were enabled by pianos equipped with motion sensors, such as the Disklavier, following their introduction to the United States in the 1980s. Repp (1996), for example, identified a range of individual expressive strategies and shared tendencies in performances of Schumann’s *Träumerei* by 10 student pianists. Responding to existing hypotheses (principally those of Clynes, 1987; Gabrielsson, 1987; Sundberg, 1988; Friberg, 1991; Todd, 1992), he found that dynamic profiles were stable and replicable between performances, though not as reliable as timing patterns; dynamic microstructures tended to reflect the hierarchic phrase-grouping of the music; and dynamics tended to increase with increases in pitch and tempo.

Repp has also contributed significantly to performance-analytical research into dynamics. Repp (1999b), for example, investigated the expressive use of dynamics in the first bars of Chopin’s Etude in E major, op. 10, no. 3 in 115 recorded performances, identifying widely shared norms of expressive interpretation, alongside individual patterns that were found to be at least as diverse as those of timing in a sister study (Repp, 1999a). More recently, Kosta et al. (2018) conducted a large corpus analysis, investigating responses to dynamic markings in 2000 recordings of 44 Chopin Mazurkas. The nuanced findings of this study—including the contingency of dynamic levels on local and large-scale context, and the positioning of dynamic markings relative to one another—epitomise the progress made in the field since the relatively basic and non-specific findings of Geringer and Breen. Considerations of style in performance analysis and the relation of dynamics in recordings to analytical readings of the score, meanwhile, has remained the province of musicological writing on performance (see for example Cook, 2013; Fabian, 2015; Leong, 2019). Constructive bridging between these methodological camps would be of benefit to future performance-analytical research into dynamics.

Of the three strands identified, most research has been dedicated to the modelling of dynamics. A range of methodologies and aims have emerged within this sub-discipline, including regular overlap with those of the studies outlined above. Advances in expressive modelling have been summarised in a number of surveys (Widmer and Goebel, 2004; Kirke and Miranda, 2009; Cancino-Chacón et al., 2018). Regularly cited examples in this field are the KTH rule system, proposing a large number of rules that can be synthesised and configured to emulate a wide variety of performance styles (Sundberg et al., 1982; Friberg et al., 2006), and Todd’s motion-based modelling system (Todd, 1992). Other strategies include the use of cubic Bezier curves to model distinctions between micro- and macro-dynamic profiles (Berndt and Hähnel, 2010); linear-basis models to explore the relationships between dynamic markings and performer contributions (Grachten and Widmer, 2012); and non-linear neural networks, trained on large quantities of existing performance data to gain insights into which aspects of a piece influence its performance (Cancino-Chacón and Grachten, 2015). The most recent research by Rhyu et al. (2022), meanwhile, uses variance autoencoder to generate expressive performances from latent score features by stochastic means.

While the majority of modelling work, including that detailed above, has focused on piano performance, recent research has branched out to explore dynamic modelling in performances by guitarists (Siquier et al., 2017), saxophonists (Lin et al., 2019), and violinists (Ortega et al., 2019), with an emphasis on the challenging physical limitations of such investigations, compared with the relatively straightforward (though nonetheless complex) key-pressing action and data retrieval possibilities afforded by the piano keyboard. Dynamic modelling of symphonic music has also been attempted by Cancino-Chacón et al. (2017) using ‘basis-functions’ of the notation in combination with non-linear neural networks to predict the dynamic results of recorded performances.

The concept of musical accents is also of relevance to this discussion. Accents can be divided into those that are immanent and those that are performed (Lerdahl and Jackendoff, 1983; Parncutt, 2003). The former are perceptual accents that derive from the inherent structure of the score, without performance variation (Thomassen, 1982; Huron and Royal, 1996). These structural aspects may be used as cues for possible changes in dynamics, assuming a connection between immanent accents and the performance (Drake and Palmer, 1993). Performed accents are those that are introduced by the performer via changes in dynamics, timing, or articulation. In this study, we regard a performed accent as a local emphasis on a note within a melody in relation to one or more surrounding notes. We also assume that the accent has a local connection to the score, that is, to the local pitch curve, the local rhythmic pattern, or the metrical position within the bar. For an overview of accent perception research, see Müllensiefen et al. (2009).

A similar approach to the current study was pioneered by Müllensiefen et al. (2009), who made predictions of perceived accents in 15 pop melodies using a set of 38 features extracted from the score. These predictions were based mainly on local features, such as rhythmic patterns, pitch patterns, and metrical position. These features were used as one of the sources for selecting features, first in Friberg et al. (2019), and then in the current study.

Of particular relevance to the current study is the modelling of immanent accents by Bisesi and Parncutt (2010), further theorised and tested in experimental rating contexts by Bisesi et al. (2019) and Friberg et al. (2019). Although these papers do not focus explicitly on dynamics, the latter provides the methodological basis for the experiment detailed in this paper, as well as the foundation for the corpus of melodies selected. In our paper, the expressively neutral presentation of melodies for audience rating of immanent accent salience in Friberg et al. (2019) is translated into a performance setting, whereby performers are required to play melodies that have been stripped of such markings expressively according to their personal taste and musical judgement.

Our central aim is thus to probe the planned, encultured, and instinctive strategies of the performers in relation to the temporal, metric, phrasal, and pitch-related features of the melodies, with an explicit focus on dynamics. We aim furthermore to expand research in this field by analysing a diverse corpus of melodies from a range of styles and with an expanded number of pianist participants, thereby assessing the influence of various feature groups on the expressive use of dynamics in different repertoires, and the unique expressive profiles of individual performers. Assessing the value and future applications of our novel methodology constitutes a secondary aim, whose rationale we address below.

## Music corpus

### Selection

In line with the perceived accent experiment of Friberg et al. (2019), a varied corpus of Western Art Music melodies was selected, representing “Baroque,” “Romantic,” and “Post-tonal” styles (see Table 1). This guaranteed a variety of musical features relating to tempo, rhythmic patterning, rhythmic periodicity, melodic contour, and global structure. We chose, as in the previous experiment, to focus on monophonic melodies, thus avoiding the more complex interaction between melody and other musical elements.

**Table 1:** List of all melodies used in the experiment.

Number	Style	Composer and piece	Bar numbers
1	B	BARBELLA, Sonate Gimo 18 for 2 mandolins and bass, 2nd Mvt.	(mandolin 1, without refrains)
2	B	BONONCINI, Il trionfo di Camilla regina dei Volsci, Aria of Camilla, “Mi lusingo”	-
3	B	CARISSIMI, Cantata, È bello l’ardire	1–9
4	B	MARINI, L’Albana, sinfonia breve	12–21
5	B	PERGOLESI, “Luce degli occhi miei”	1–15
6	B	RAMEAU, Les Cyclopes	1–14
7	B	SAULI, Partita II for mandolin	1–10
8	B	SCARLATTI, Sonata in D minor K 213	1–8
9	B	TORELLI, Concert in E minor, Op. 8 No. 9	1–14
10	B	VITALI, Toccata for solo cello	1–11
11	R	ALBÉNIZ, Rimas de Bécquer	1–8
12	R	BELLINI, I Capuleti e i Montecchi, Act I, Scene IV, “Oh quante volte oh quante”	3–10
13	R	BRAHMS, Intermezzo, Op. 117 No. 1, inner melody – rh	1–8
14	R	DONIZETTI, Linda di Chamounix, “O luce di quest’anima”	10–25
15	R	NORMAN, Svar	1–18
16	R	MAHLER, Simphony No. 4, IV Mvt., “Sehr behaglich,” voice	13–25
17	R	MASCAGNI, Cavalleria Rusticana, Intermezzo sinfonico, vl. I	1–10
18	R	MASSENET, Dèpart	2–10
19	R	MENDELSSOHN, Lieder ohne Worte, Op. 19 No. 4	9–17
20	R	ROSSINI, Guillaume Tell, Symphonie	(No. 7, Andante, from mark 8 to mark 9)
21	PT	BARTÓK, String Quartet no. 4, mvt I	1–13
22	PT	BERG, Lyrische Suite, II, Andante amoroso	1–8
23	PT	BOULEZ, Douze Notations, 3	1–8
24	PT	MAXWELL DAVIES, Five Little Pieces, no. 2	1–14
25	PT	MESSIAEN, Prelude no. 7	1–11
26	PT	SCHOENBERG, Pierrot Lunaire Op. 21, II, “Colombine,” voice	1–12
27	PT	SLOMINSKY, 50 miniatures, no. 2	1–15
28	PT	STOCKHAUSEN, Aquarius, Tierkreis	1–23
29	PT	VARÈSE, Offrandes for soprano and chamber orchestra, II, “La croix du sud,” voice	8–20
30	PT	WEBERN, Canon, Op. 16 No. 2 for voice and clarinet, voice	2–7

B=Baroque, R = Romantic, PT = Post-tonal.

The number of melodies in the new experiment was reduced from 60 to 30, owing to the time restraints of the recording and the preparatory commitment of the pianist participants. The distribution of melodies within each style was reduced from 20 to 10. As many melodies as possible were included from the previous experimental corpus to allow for analytical comparison, though this remains limited in the current study. It was, however, necessary to consider the pianistic suitability of the melodies.

Each of the original melodies was tested for playability and difficulty with respect to tempo, technical demands, configuration of white and black keys, and ease of fingering. A large number of melodies, particularly those for voice or non-keyboard instruments, were deemed unsuitable on these grounds. Ultimately, 8 melodies were selected from the original Baroque corpus, 10 from the original Romantic corpus, and 4 from the original Post-tonal corpus. Two new Baroque melodies and 6 Post-tonal melodies were selected on the basis of their playability and stylistic contribution to the existing corpus. In total, 9 of the melodies were for keyboard instruments (piano and harpsichord), 9 were instrumental, and 12 were vocal.

### Presentation

In accordance with the ‘neutral’ MIDI presentation of melodies in the perceived accent experiment, all expressive markings were removed from the melodies in the new corpus. All titles and composer information were also removed. Each melody was allocated a fixed number from 1 to 30. Metronome marks were given for each melody. These were either maintained from their use in the perceived accent experiment or taken from the composer’s own indications. As with the perceived accent experiment, for pieces without a preordained metronome mark a tempo was determined according to the style and period of the piece and/or its expressive tempo direction (e.g., *Andante* = 75 b.p.m.). Each melody was formatted to make it pianistically legible. This involved standardising metrical beaming, clearly spacing and distributing bars and bar lines, and transposing all melodies to within the range of the treble clef. Notation in the range of the treble clef was designed to encourage performers to play all melodies with the right hand, thereby avoiding any inconsistency in right- and left-hand technique among the participants, and the potential for interpretation to be affected by the mirrored characteristics of the hands (i.e., thumb at the “top” of the left hand, little finger at the “top” of the right hand). Performers were given the option to use paper or digital copies during their recorded performances. All notation was produced in a standard formatting via MuseScore 3.

Our rationale for presenting the melodies in this manner was threefold: (1) to investigate whether pitches that are perceived to be more important when presented without dynamic alterations are afforded corresponding emphases by means of the expressive use of dynamics. In other words, to investigate the relationship between immanent and performed accents of different kinds as they relate to certain structural features of the score involving pitch, phrasing, duration, rhythm, and metre. (2) to allow for comparison of expressive performance practice across periods in relation to these same structural features. Historically speaking, dynamics and other forms of expression in earlier music tend to be implicit and are rarely directed in the score, whereas they tend to be explicit and often highly prescribed in later music, especially that of the twentieth and 20th and 21st centuries. By eliminating these markings across our corpus, we therefore aim to investigate fundamental expressive tendencies relating to a wide range of structural features, while avoiding the subjective complexity involved in analysing the stylistic interpretation of prescribed markings. This is not to negate or ignore the stylistic awareness of the pianist, but rather to bring it into more equitable focus. (3) by investigating the expressive performance of melodies without dynamic markings, we place our research in dialogue with investigations of the interpretation of prescribed markings in different musical corpora (see for example Kosta et al., 2018), inviting future comparative research between instinctive/personal and prescribed dynamic interpretation within and between different repertoires.

## Performance experiment

### Participants

Participants were recruited via email from the School of Music at the University of Leeds, Leeds Conservatoire, and the Department of Music at the University of York. Twenty participants were recruited in total (11 females and 9 males; 14 from the United Kingdom, 5 from China, and 1 from Hungary; 17 aged 18–25, 1 aged 31–40, 1 aged 41–50, and 1 aged 50+). All participants were expected to have at least an undergraduate level of first-study competence in piano performance and a grounding in music theory and style. Twelve were undergraduate music students, 5 were masters music students, and 3 were PhD candidates. The pianistic level of the participants ranged from ABRSM Grade 8 to working professional pianists two of the PhD candidates, with participants citing a wide range of performing and teaching experience.^1^

Our rationale for using a relatively large number of participants was to identify commonalities of approach and to attempt to reduce data noise. By financial and practical necessity, we were only able to recruit non-professional student pianists from within higher education settings, in addition to the two professional PhD candidates. The student pianists were, however, of a high standard—as identified in preliminary questionnaires—pursing first-study piano in undergraduate and postgraduate performance courses at well-respected higher education music institutions. The task was also carefully designed by the professional pianist author of the study to suit the technical levels of the participants (see Section 2.1), with single lines rather than complex textures to be performed and ample time provided in which to prepare. There was, nonetheless, an element of experimentation involved in this methodology, which proved to be effective in some regard, though challenging to moderate in others. Whether or not the use of a large dataset of 20 predominantly non-professional pianists—typical of financially viable recruitment from higher education music institutions and conservatoires—has value or may be of value for future research therefore constitutes one of the secondary experimental aims of the study.

### Rubric

Participants were given digital copies of the melodies a week in advance of their scheduled recording session. They were asked to play the melodies according to their ‘personal taste’ with ‘all matters of dynamics, articulation, and phrasing left up to the performer’. They were requested not to use sustaining pedal. In certain instances, such as leaps exceeding the span of one hand, the left hand was permitted to assist the right, in order to convey the desired expression (e.g., a legato connection). They were told that the performance should attempt to adhere to the given metronome mark, though expressive fluctuation from this pulse was permitted as and when it was deemed appropriate. They were told to prepare the melodies thoroughly, including working out necessary fingerings. They were also permitted to make annotations to their scores, which they could refer to during the recordings should they choose.

### Procedure

The participants performed the melodies on a Disklavier DKC-800 in the School of Music at the University of Leeds. MIDI data was recorded directly to a USB drive. A simultaneous audio recording was made using two stereo microphones placed directly behind the pianists, though this back-up source was not used for analysis. Each participant was allocated a two-hour recording session, with no-one exceeding this duration. Each participant was asked to perform the remaining melodies in a uniquely randomised order, beginning each time with Melody 1. Once all of the melodies had been recorded, Melody 1 was repeated as a means of assessing the consistency of the performances. Participants were encouraged to repeat melodies that were deemed technically unsatisfactory, either by the participant themselves or by the experiment leader. Limited errors in pitch were permitted, with repeat recordings reserved for significant technical mishaps, unintentional pauses, or clear errors in meter or rhythm. While the number of repetitions was not recorded, certain pianists required many more retakes than others. This generally correlated with their level of experience, as indicated by the initial questionnaire, and their level of preparation, as subjectively determined by the experiment leader. Prior to recording each melody, participants were given the relevant metronome beat as a reference point. Following the experiment, they were asked to complete a follow-up questionnaire, detailing their approach to the task and an assessment of its difficulty.

### Questionnaire results

The participants were asked to rate the difficulty of the task on a five-point Likert scale, ranging from “easy” to “very difficult.” The mean response was 3 with a standard deviation of 0.73, suggesting a suitable level of difficulty for the standard of the pianists chosen. When asked to specify difficulties encountered in the task, 16 of the participants cited realising certain rhythms, particularly those found in the post-tonal examples. Six of the participants reported challenges with tempo. This chiefly referred to more active melodies with higher tempi, though Participant 2 (P2) also noted how ‘some of the metronome marks felt a little unnatural, which meant that greater finger control was required (generally to keep the speed down)’. Four of the participants cited difficulty in identifying the style of the melodies (P3, P4, P9, P12). Four of the participants cited difficulty in realising accidentals, in particular double flats in certain post-tonal examples (P7, P12, P13, P18). Two of the participants cited concentration during the recording session, with P3 noting how ‘having not memorized most of the melodies, it became tricky to keep up the level of sight-reading as I came to the melodies played later on in the recording session (essentially, when I started to get a bit more brain-tired)’, and P12 noting how ‘I found it difficult with the longer extracts to play through without thinking of making any mistakes’. The remaining difficulties cited related to technique (P2, P3), fingering (P3, P16), and lack of harmonic context (P2).

The participants were also asked to rate their familiarity with the style and period of the melodies on a five-point Likert scale, ranging from ‘no awareness of style and period’ to ‘awareness of style and period for every melody’. The mean response was 3.45 with a standard deviation of 0.89, suggesting a fairly high level of stylistic awareness among the participants. This was further reflected in reports of the participants’ expressive approach to performing the melodies, with 9 of the participants citing awareness of style as contributing to their expressive approaches (P2, P3, P5, P8, P9, P13, P14, P16, P17). For example, P10 noted how ‘I try to figure out what style or era the melody is from’; P9 noted how they ‘recognised style or genre’; P17 noted how ‘I tried to connect the melodies to a musical style or era, then use elements of that in the way that I played’; and P12 noted how ‘I could also sometimes recognise the historical period of the extract and this helped in my interpretation and expression’. Several participants also cited difficulty in approaching melodies that were perceived as more ‘contemporary’ or ‘modern’ in comparison with those identified as ‘Baroque’, ‘Classical’, or ‘Romantic’. For example, P2 noted how ‘Baroque or Classical pieces were approached in a conventional manner, but the more modern melodies had to be considered in terms of “should high be loud or quiet”’; P16 noted how ‘for more dissonant and rhythmically complex melodies, I found it more difficult to add expression and tried to just vary dynamics and articulation’; and P13 noted how ‘with the more contemporary music I think I could have used more contrasting dynamics and articulation to make my interpretations sound convincing.’ Meanwhile, P14 noted how ‘not having any […] contextual knowledge about the composer/genre it was tricky to know what would work’, and P5 noted that since ‘the melodies were not defined in terms of period [they] could be played in several different styles’.

Half of the participants associated a rise in melodic contour with an increase in dynamic (i.e., high-loud; P2, P3, P4, P9, P10, P14, P16, P18, P19, P20). For example, P14 cited a ‘general rule’ of ‘getting louder to the top of a phrase and *vice-versa*’; this was indicative of the comments of the other participants. As noted, P2 found this more challenging in the context of melodies with more disjunct melodic lines.

Seven of the participants cited ‘instinct’ or ‘freedom’ as a factor in their expressive approach. For example, P2 noted how, ‘I played them through first, going on instinct […] Because of the lack of phrasing and articulation, it was possible to have a lot of fun’; P11 noted how ‘I performed them with […] the freedom of creating my own style’; and P20 noted how one should ‘sing in your heart’ when playing.

A number of participants cited tempo (P10, P12, P18), rhythm (P9, P18), and implied harmony (P3, P9, P14) as deciding factors. Two participants also cited structural context, or lack thereof, as significant (P3, P8). For example, P3 noted how ‘I tried to appreciate the melody as the entire song - this would have made some of the melodies more expressive perhaps than if they were in a denser textual context, but I believe that is how a melody should be approached in any context regardless’, and P8 noted how ‘I try to […] guess what it would’ve sounded like in a full piece’. Finally, two participants cited dissatisfaction with aspects of their expressive performance, with P7 noting how for ‘some little parts I did not do well’, and P6 noting how ‘I should have used more dynamics’.

### Basic processing and analysis of performance data

For processing the resulting MIDI files and computing the features, we used the software Director Musices (DM; Friberg et al., 2006). Using a custom-made script in DM, the performed MIDI files were aligned with the corresponding score, automatically taking into account errors relating to one-note contexts such as individual missing/added notes or wrong pitches. These notes were marked in the score and omitted from the modelling. In total, 246 were notes omitted, corresponding to 0.72% of all notes. These were principally missing or extra notes in the performance.

We calibrated the system by measuring the relation between MIDI velocity and the sound level for the specific Disklavier piano used. Using these measured velocity curves, the velocity numbers in the performed MIDI files were translated into sound levels. The resulting sound level was normalised for each song and participant. This removed eventual bias in case the participants differed in terms of their overall dynamic level.

The first step was to form a relevant average measure of the performed sound level across the participants. In this study, we took the mean value across the participants, using a special procedure to ensure that each participant contributed to the reliability of the average measure. For estimating the reliability of the average measure, we used Cronbach’s alpha (CA). CA is the same measure as the intra-class correlation ICC case 2 (see McGraw and Wong, 1996). For illustrating the variance among the participants, we used the average pair-wise Pearson’s correlation across all participants. The resulting values are shown in Table 2 for all melodies and for the different styles. The relatively high values of Cronbach’s alpha supported the use of average values for the subsequent modelling. Formally, the estimation of CA assumes an underlying normal distribution. However, the estimation error for a non-normal distribution is negligible for large sample sizes (as in this case) and the eventual bias is negative (Sheng and Sheng, 2012). Therefore, a high alpha value is always an indication of a good reliability regardless of underlying sample distribution. A value above 0.8 is considered a very good reliability, which is exceeded for all melody groups in Table 2.

**Table 2:** The agreement among the participants in terms of the Cronbach’s alpha and the mean pairwise correlations presented for all melodies and for the different styles.

Melodies	Number of notes	Cronbach’s alpha	Mean pairwise correlation
All	1701	0.880	0.291
Baroque	730	0.872	0.277
Romantic	541	0.893	0.311
Post-tonal	430	0.870	0.287

**Figure 1:**
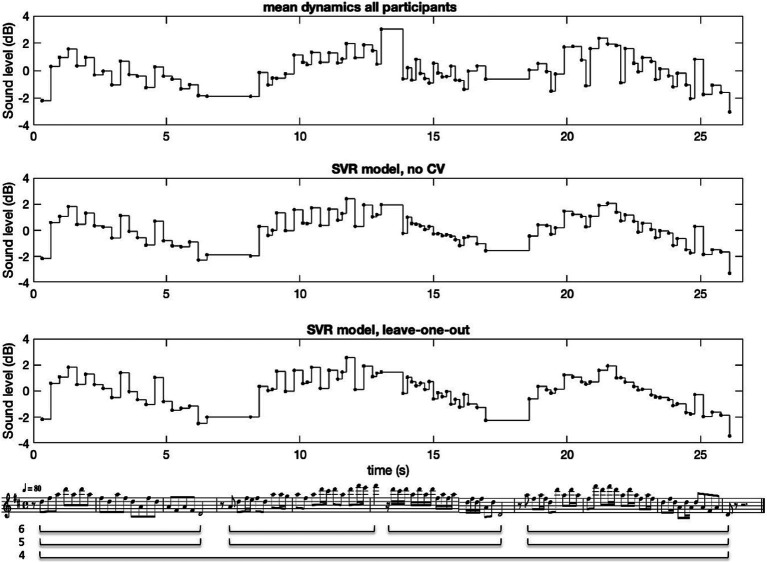
The average dynamics and the model result for Melody 10, Toccata for solo cello by Vitali. The three phrase levels are shown under the score.

We also tested an exclusion procedure to improve the reliability of the average measure (see also Elowsson and Friberg, 2017; Friberg et al., 2019), whereby participants were excluded one-by-one and the new CA value was compared with the CA computed for all participants. If CA increased when a participant was excluded, the procedure was repeated for the remaining participants until the CA did not increase any further. Using this procedure, Participant 1 was excluded from the computation of the mean value. This participant was also found to have comparative difficulty in performing the different melodies, as observed by the experiment leader. The result of this exclusion was, however, a rather modest improvement of CA = 0.883 and mean pair-wise correlation = 0.313 across all styles. For the sake of simplicity, we therefore chose to disregard this selection procedure, using the full average across all 20 participants in the following computations. An example of the resulting average dynamics curve for Melody 10 is shown in Figure 1.

In order to further validate the use of the average for the analysis, both authors (of which one is a professional pianist) listened informally through all of the examples with the average dynamics amplified so that the note-to-note variations were more clearly perceived. Both authors found that all 30 generated performances of the average made sense from a musical point of view, and that there was not any single tone that stood out in a negative sense. These sound examples can be heard online.^2^

## Dynamics modelling

To a large extent, the modelling procedure follows the same methodology developed in Friberg et al. (2019), beginning with a set number of features that are used to predict the dynamics obtained in the experiment. The developed models are then used to examine the role of the different feature groups relating to musical aspects such as rhythm and pitch, as well as the specific expressive approaches of the different pianists.

### Features

We started by including all of the features that were developed for modelling immanent accents in melodies (Friberg et al., 2019). Most of these features have a local character with a maximum context of five notes (see Table 3). The features were divided into five subgroups. The *pitch contour* features consider the relation of pitches in a small context, such as melodic leaps, peaks, or arpeggios. The *tempo* features focus on the duration of notes. The *timing pattern* features consider rhythmic relationships in localised contexts, such as a short note between longer notes. The *simple phrasing* features look at notes before and after rests. Finally, the *meter* features look at the metrical position of each note in the bar. For a more detailed description of these features, see Friberg et al. (2019).

**Table 3:** The local features used in the study together with a short description.

Category	Nr	Name	Description
*Pitch contour*	1	f0_pos_dist_mean_2bar	Positive distance to running pitch mean over the length of two bars (semitones)
	2	f0_neg_dist_mean_2bar	Negative distance from mean of 2 bars (semitones)
	3	f0_pos_dist_mean_1bar	Positive distance from mean of 1 bar (semitones)
	4	f0_neg_dist_mean_1bar	Negative distance from mean of 2 bars (semitones)
	5	f0_bef_pos_leap_p (_log)	First note of positive leap (1/0 or log leap size)
	6	f0_bef_neg_leap_p (_log)	First note of negative leap (1/0 or log leap size)
	7	f0_aft_pos_leap_p (_log)	Second note of positive leap (1/0 or log leap size)
	8	f0_aft_neg_leap_p (_log)	Second note of negative leap (1/0 or log leap size)
	9	f0_aft_leap2_log	Third note in a four-note context with leap in the middle and with an up-down-up or down-up-down pattern (log leap size)
	10	f0_bef_leap2_log	The same as above but marked on second note (log leap size)
	11	f0_pos_peak_p (_log)	Positive peak in three notes, one before one after (1/0 or log leap size)
	12	f0_neg_peak_p (_log)	Negative peak in three notes, one before one after (1/0 or log leap size)
	13	f0_pos_peak2_p	Positive peak in four notes, two before one after (1/0)
	14	f0_pos_peak3_p	Positive peak in five notes, three before one after (1/0)
	15	f0_first_arp_up_p	First note in upward arpeggio, two up-leaps preceded by any other interval (1/0)
	16	f0_last_arp_up_p	Last note in upward arpeggio (1/0)
	17	f0_first_arp_down_p	First note in downward arpeggio (1/0)
	18	f0_last_arp_down_p	Last note in downward arpeggio (1/0)
*Tempo*	19	dr_ndr	IOI (ms)
	20	dr_very_short_note	Very short notes (weight 0–1)
*Timing patterns*	21	dr_short_before_p (rel,_log)	One long note after short (1/0, relative, log)
	22	dr_short2_before_p	One long note after two equally short notes (1/0)
	23	dr_short3_before_p	One long note after three equally short notes (1/0)
	24	dr_short_after_p	One long note before short (1/0)
	25	dr_first_short_p	First of at least two short notes (from punctuation) (1/0)
	26	dr_longest_five_p (_w)	Longest in the middle of five notes (1/0 or gradual weight)
	27	dr_short_between_long_p	A short note between two longer notes (1/0)
	28	dr_long_after_p	Short note before long (1/0)
*Simple phrasing*	29	ph_rest_before_or_first	Note after rest or the first note
	30	ph_rest_after_or_last	Note before rest or the last note
*Meter*	31	beat0	Sub-beat (1/0)
	32	beat1	Beat or tactus level (1/0)
	33	beat2	Half bar or bar (1/0)
	34	beat3	Bar or 2 bars (1/0)

The parentheses in the feature names indicate alternative formulations. The extension “_p” indicates a binary variable; any other extension indicates a varying weight value.

The recorded dynamics curve is a summation of many different aspects of performance (Friberg and Battel, 2002). Therefore, it was considered necessary to also include features with a larger context than those used in Friberg et al. (2019), in order to model more long-term variations in the performances, in particular those relating to phrasing. To achieve this, we included a selection of features derived from the set of performance rules previously defined for music performance (Friberg et al., 2006; see Table 4). These features were divided into two subgroups.

**Table 4:** The rule-based features used in the study together with a short description.

Category	Nr	Feature name	Description
*Automatic rules*	35	ru_chromch_dsl	Chromatic charge – emphasise close pitch regions
	36	ru_hiloud_dsl	High-loud – the higher the pitch the louder
	37	ru_durcont_dsl	Duration contrast – the shorter the note the softer
	38	ru_punct_dro	Punctuation – small melodic groups are recognized and the final note in each group is articulated with a small micropause
	39	ru_punct_first	Punctuation – first note of each group
*Phrasing rules*	40	ru_ph4_dsl	Phrase arch level 4 (highest level) – the whole melody
	41	ru_ph4_start	Phrase arch level 4 – mark the first note
	42	ru_ph4_end	Phrase arch level 4 – mark the last note
	43	ru_ph5_dsl	Phrase arch level 5 – the major phrase level
	44	ru_ph5_start	Phrase arch level 5 – mark the first note
	45	ru_ph5_end	Phrase arch level 5 – mark the last note
	46	ru_ph6_dsl	Phrase arch level 6 – the subphrase level
	47	ru_ph6_start	Phrase arch level 6 – mark the first note
	48	ru_ph6_end	Phrase arch level 6 – mark the last note

The *automatic* rules, as the name suggests, can be computed from a normal score without any extra information (as with all of the low-level features in Table 3). They consist of a small selection of rules that might be relevant in this context for modelling dynamics. The *chromatic charge* rule was developed specifically for application to atonal melodies. Its principle dictates that areas where notes are close together in pitch are emphasised, while areas with larger leaps between notes are less emphasised (Friberg et al., 1991). The *high loud* rule emphasises higher pitches and deemphasises lower pitches. The sound level variation is linear as a function of pitch and is normalised around the average pitch across all notes in the melody (Sundberg et al., 1982; Friberg, 1991). The *duration contrast* rule deemphasises relatively short notes by making them softer (Sundberg et al., 1982; Friberg, 1991). The *punctuation* rule is an attempt to automatically find small groups of notes that belong together (Friberg et al., 1998). These groups are performed by adding a micropause after the last note of each group and by lengthening its duration. Here we used the length of the micropause and the first note of each group as features.

The *phrasing* rules apply an arch-like curve for dynamics and tempo across phrases on different hierarchical levels (Friberg, 1995). Analysing the phrase structure in a melody by automatic methods is a challenging task. Therefore, these rules require the phrase structure to be marked manually in the score. To do this, the authors marked three different levels. Level 4 corresponds to the whole melody, level 5 to certain longer phrase divisions, and level 6 to the smallest phrase units.^3^ One example is provided in Figure 1. The *phrase-arch* rule contains several additional parameters that were chosen as the best fit for a set of analysed performances of Schumann’s *Träumerei* (Repp, 1992). The features included both the whole dynamics curve and the first and the last note of each phrase separately for all three levels. Introducing the *phrasing* rules was considered necessary in order to account for the large phrasing curves that sometimes appeared in the performances. However, this process is somewhat problematic since it introduces both the subjective marking of the phrases as well as making it more cumbersome to apply to new melodies.

We chose not to include features related to the implicit harmony. It would introduce one more subjective factor similar to the phrasing since the harmonic analysis needs to be done manually. The effect of harmony was not as evident in the data as the phrasing, although it was indeed mentioned by the participants as a factor that influenced the dynamics. This could be investigated in a future study.

For some of the features, two or three alternative versions were defined, usually with one simple version in the form of a binary feature (simply 0 or 1) and another version with a gradual increase in proportion to some contextual parameter (thus, a varying number). For example, the feature ‘first note of a positive leap’ was formulated as a binary variable (_p) and a gradual variable, depending on the leap size (_log). For all features with alternative definitions (as specified in Table 3) only those with the highest correlation to mean dynamics were selected in the final feature set. This left 48 features which were then used in the subsequent analysis.

### Prediction methods and training

For the prediction of the performed dynamics, we used both multiple linear regression (MLR) and support vector regression (SVR). MLR was chosen as it gives very detailed analysis of the contribution of each feature. However, as it is a linear combination of features, it does not model interactions between the features. For this purpose, we used SVR, which was expected to perform better than MLR as it includes interactions. Both models were computed in Matlab, v 2022b. MLR was applied using the built-in function ‘regstats’ and the SVR was computed using the LIBSVM library version 3.22 for Matlab (Chang and Lin, 2011). For the SVR, a radial basis function was used as the kernel and the model parameters C and gamma were optimised using a grid search. The first evaluation method was 10-fold cross-validation, where the final result was the average over 10 random repetitions. The second evaluation method was leave-one-out, in which the model was trained on all melodies but one, and then evaluated on the omitted melody. This was repeated for all melodies and the results were averaged. The advantage with the latter method is that it corresponds better to a real situation in which the model is applied to an unknown piece of music.

### Overall prediction results

The results of the MLR and SVR predictions are shown in Table 5 both for 10-fold and leave-one-out cross validation. As expected, the best results were obtained by the SVR method, explaining about 64% or 61% of the variation in dynamics, depending on the cross-validation method. Thus, the overall prediction of dynamics was rather modest. However, it was deemed sufficient for pursuing the more detailed analysis of the different feature groups and individual variations below. Note that the 10-fold method varies somewhat due to the random selection of folds, while the leave-one-out method is deterministic and always obtains the same result if the model is rerun.

**Table 5:** Overall prediction results.

	*R*^2^ 10-fold CV (%)		*R*^2^ leave-one-out (%)	
*Features*	*48*	*34*	*48*	*34*
MLR	58.4	58.7	55.8	56.4
SVR	64.2	65.9	61.0	63.6

The number of features is either the full set (48) or the reduced set after feature reduction (34).

As an alternative to using the average across pianists, we also tested the application of the MLR model using the median across pianists. The purpose was to see if there might be remaining outliers that affected the overall measure and thus the prediction. Using the model prediction in this context is justified since all the defined features are related to the score in a consistent way; thus, everything that can be modelled can be considered as related to the score and not to random variations. The model results for MLR using the median were 58.4% for 10-fold cross-validation (using the average it was 58.4%; see Table 5) and 56.2% for leave-one-out (using the average it was 55.8%) using 48 features. This modest improvement (or no change) was not considered sufficient for using the median values instead as it would only result in marginal differences for the subsequent analysis of the impact from the feature groups.

A feature reduction was made using sequential feature selection. The SVR prediction with leave-one-out was used for the evaluation in all steps. The main idea was to remove features that contribute negatively to the final cross-validated result. In the first step, an SVR was computed for each case when one feature at a time was removed. The results were compared to the SVR result with all features. The feature that made the most negative contribution was removed. The whole procedure was then repeated for the remaining features. This procedure was rerun until there were no more features contributing negatively (see also Maldonado and Weber, 2009). Fourteen of the 48 features were removed on this basis. Although a significant number of features contributed negatively, there were only modest improvements (see Table 5). Since it was optimised on SVR with leave-one-out, this case yielded the largest improvement (2.6%), as expected. However, the MLR result also improved somewhat. In light of this slight improvement, we decided to use the original set of 48 features for the subsequent analysis.

We also applied MLR without cross validation for all features. This resulted in an overall *R^2^* of 61.3% that was just slightly higher than the cross-validated results (not shown in Table 5). Therefore, we concluded that the over-fitting in the MLR model was modest and that a more detailed analysis of the features could be performed in the next section.

An example of the resulting dynamics profiles for the measured average (top graph) and the SVR model (graph 2 and 3) is shown in Figure 1. In the measured average a clear phrasing pattern across the three main phrases can be observed (phrase level 5). In the first phrase, the local dynamic accents coincide with local pitch peaks. In the second phrase, there is a clear global peak on the top note in the middle of the phrase, and local accents on the quarter-note beat subdivision, sometimes coinciding with relatively long notes. That the variation in dynamics is coupled to the structure in a similar way could also be observed for the other melodies. Together with the relatively high Cronbach’s alpha, this is an indication that the average across pianists is a relevant measure to investigate. The SVR model captured these variations to various degrees, as seen in graph 2 and 3. The overall phrasing is nonetheless rather well modelled, and the accent patterns are noticeably captured for some parts of the phrases (mainly phrase 1 and the first half of phrase 2).

### Correlation analysis

As a first test, we computed correlation between each feature and the average dynamics of the participants (see column 3 in Table 6). We used either the point-biserial or the Pearson’s correlation depending on whether the feature was binary or gradual. The significance levels indicated in the table should be taken with some caution since they have not been compensated for multiple testing. They can, however, serve as an indication of the importance of each feature. As seen in the table, a majority of the features are correlated with the dynamics. The highest correlation was between the *high loud* rule and the dynamics (*r* = 0.62). This correspondence between pitch and dynamics was also evident when manually inspecting the dynamics curves and reflected in the participant feedback, with half of the pianists citing an increase in melodic contour as indicative of the need for an expressive increase in volume (see section 3.4). One notable exception was the meter features, which seem to be quite weakly correlated to the dynamics, thus giving a first indication that the metrical structure is not emphasised in this data. This observation was further supported by the fact that only two of the participants cited rhythm as a factor in their expressive approach (see again section 3.4).

**Table 6:** The resulting correlations between features and the mean dynamics (column 3).

		Correlation	Multiple linear regression		
Category	Feature	*r*	beta	*sr*	Value of *p*
*Pitch contour*	f0_pos_dist_mean_2bar	0.52***	0.142	**0.053**	0.001***
	f0_neg_dist_mean_2bar	−0.51***	−0.058	0.022	0.160
	f0_pos_dist_mean_1bar	0.48***	0.057	0.022	0.152
	f0_neg_dist_mean_1bar	−0.47***	−0.044	0.017	0.257
	f0_bef_pos_leap_p	−0.11***	0.045	0.034	0.027*
	f0_bef_neg_leap_log	0.19***	−0.003	0.002	0.901
	f0_aft_pos_leap_log	0.26***	0.022	0.013	0.393
	f0_aft_neg_leap_log	−0.24***	−0.027	0.016	0.286
	f0_aft_leap2_log	0.01	0.044	0.033	0.030*
	f0_bef_leap2_log	0.08***	0.035	0.028	0.063
	f0_pos_peak_log	0.29***	−0.069	0.032	0.038*
	f0_neg_peak_log	−0.22***	−0.060	0.035	0.022*
	f0_pos_peak2_p	0.31***	0.011	0.005	0.761
	f0_pos_peak3_p	0.31***	0.057	0.030	0.048*
	f0_first_arp_up_p	−0.13***	−0.028	0.025	0.102
	f0_last_arp_up_p	0.14***	0.005	0.004	0.788
	f0_first_arp_down_p	0.11***	0.012	0.011	0.475
	f0_last_arp_down_p	−0.09***	0.034	0.030	0.049*
*Tempo*	dr_ndr	−0.03	−0.063	**0.043**	0.005**
	dr_very_short_note	−0.05*	0.016	0.014	0.358
*Timing patterns*	dr_short_before_p	0.13***	0.088	**0.050**	0.001**
	dr_short2_before_p	0.11***	0.029	0.016	0.291
	dr_short3_before_p	0.05*	0.001	0.001	0.963
	dr_short_after_p	0.13***	0.049	0.023	0.141
	dr_first_short_p	−0.08**	−0.065	**0.049**	0.001**
	dr_longest_five_w	0.12***	0.014	0.009	0.570
	dr_short_between_long_p	0.11***	0.047	0.035	0.023*
	dr_long_after_p	−0.09***	−0.044	0.036	0.019*
*Simple phrasing*	ph_rest_before_or_first	−0.19***	−0.033	0.020	0.195
	ph_rest_after_or_last	−0.26***	−0.090	**0.060**	0.000***
*Meter*	beat0	0.02	0.016	0.013	0.395
	beat1	0.06*	0.041	0.028	0.066
	beat2	0.02	0.035	0.022	0.152
	beat3	−0.03	−0.024	0.017	0.273
*Automatic rules*	ru_chromch_dsl	0.04	−0.012	0.011	0.483
	ru_hiloud_dsl	0.62***	0.340	**0.180**	0.000***
	ru_durcont_dsl	−0.04	−0.051	0.036	0.019*
	ru_punct_dro	0.13***	−0.014	0.006	0.689
	ru_punct_first	−0.26***	−0.137	**0.091**	0.000***
*Phrasing rules*	ru_ph4_dsl	0.21***	0.031	0.022	0.147
	ru_ph4_start	−0.11***	−0.022	0.017	0.270
	ru_ph4_end	−0.24***	−0.071	0.044	0.004**
	ru_ph5_dsl	0.34***	0.121	**0.063**	0.000***
	ru_ph5_start	−0.18***	0.012	0.007	0.670
	ru_ph5_end	−0.25***	0.043	0.020	0.194
	ru_ph6_dsl	0.26***	0.213	**0.072**	0.000***
	ru_ph6_start	−0.19***	−0.094	**0.061**	0.000***
	ru_ph6_end	−0.21***	0.052	0.016	0.296

The result of the multiple linear regression showing the beta coefficient, the semipartial correlation *sr*, and the *p* value (column 4–6). Significance levels *p < 0.05, **p < 0.01, ***p < 0.001.

Note that all *phrasing* rules (_dsl) are positively correlated and that both the first note (_start) and the last note (_end) of each phrasing level is negative, indicating that the first and the last notes of the phrases are played softer, in agreement with previous research (e.g., Gabrielsson, 1987).

### Feature analysis using MLR

The detailed results of the MLR Method applied without cross validation are shown in Table 6. All features are listed along with the beta-weight, the semipartial correlation coefficient *sr*, and the corresponding value of *p*. The *sr* coefficient reflects the independent contribution of each feature. The most important features, with *sr* > 0.04, are marked in bold.

In comparison with the individual correlations in column 3, substantially fewer features are significant in the MLR model. By far the strongest influence is the *high loud* rule (ru_hiloud_dsl) with *sr* = 0.180. By contrast, there is little influence from *meter* features, although the beat1 level almost reaches significance. Several of the different *phrasing* features also make a significant contribution.

### Influence of feature groups

For investigating the influence of each feature group, we used the SVR model with the 10-fold cross validation method (see Table 7). Here, we grouped all features into the main categories according to musical function. Thus, the *pitch* group contains the *pitch contour* features, the *chromatic charge* rule (ru_chromch_dsl), and the *high loud* rule (ru_highloud_dsl); the *timing* group contains both the *tempo* and *timing pattern* features as well as the *duration contrast* rule (ru_durcont_dsl); the *meter* group stays the same; and the *phrasing* group contains the *simple phrasing* features, the *punctuation* features (ru_punct_dro, ru_punct_first), and the *phrasing* rules.

**Table 7:** The individual contribution in terms of R^2^ for each feature group and style.

	Model *R*^2^ (%)	Independent contribution of each feature group (%)			
Music selection	*All features*	*Pitch*	*Timing*	*Meter*	*Phrasing*
All	64.2	37.3	2.8	0.7	12.3
Baroque	59.1	34.6	1.6	**1.3**	6.0
Romantic	**66.6**	29.5	**2.8**	0.2	**18.8**
Post-tonal	61.5	**43.6**	−0.5	1.0	7.9

The highest value in each column is marked in bold.

The independent contribution was calculated by applying the model without each group and then comparing it with the full model. Thus, it is a comparable measure to the semipartial correlation coefficient *sr* used in the MLR analysis in the previous section. Note that the sum of the independent contributions from each group is less than the overall result, indicating that the feature groups overlap to some extent. Also, there are some negative values, i.e., the model performed slightly better without these features in these cases. Although these negative values are rather small, it may indicate that there is a certain amount of over-fitting, also considering that there are a smaller number of cases (note events) when analysing only one style at a time.

Overall, the *pitch contour* (37.3%) and *phrasing* features (12.3%) obtained the highest independent contribution across styles. Presumably, a large part of the contribution from the pitch category can be attributed to the *high loud* rule, as indicated by the relatively high correlation to the performed dynamics shown in Table 6. As noted, the use of the *high loud* rule in piano performance has been observed in previous studies (Repp, 1996) and was also explicitly mentioned in the questionnaire by half of the participants (see section 3.4).

When comparing the different styles, contributions from the Romantic category stand out, having had a somewhat lower influence from the *pitch contour*, substantially higher contribution from the *phrasing*, and essentially no contribution from the *meter* features. The influence of the *timing* features was surprisingly small for all styles. This implies that rhythmic patterns may be of little importance for dynamics, at least in relation to these specific features and in the musical context of unaccompanied melodies. The lowest value for the timing group was obtained for the Post-tonal style. This may reflect the heightened rhythmic complexity of some of these examples (particularly Melodies 23 and 24), which a number of participants reported as being particularly difficult to perform. The influence of *meter* features was also rather small, though there was still some contribution, in particular for the Baroque style. *Phrasing* features were most influential for the Romantic style.

### Personal profiles

The same SVR model with the same grouping was applied again on each individual participant’s dynamics profile (see Table 8). The explained variation for the full model varies from a mere 14% for P4 up to almost 47% for P2. Note that this is about 18% lower than for the average data in Table 7 (64.2). This is an indication that the data from the individual pianists contain more noise than the average, although we cannot rule out that individual pianists may instead have used different strategies for similar passages in the score, or that they let inspiration in the moment influence the performance. The data also show that the participants varied in their approach, as manifested by the variation of the independent contribution from the different feature groups. For example, P1 (and to a certain extent P17) relied more on *timing* and *meter* features, while a number of other participants focused more on *pitch* and *phrasing* features (P2, P3, P7, P12). Other participants had more varied profiles, suggesting the influence of more diverse combinations of features. Comparing the musical levels with the model results shows that the two non-composition PhD students (also professional pianists) were comparatively well predicted by our model (P1 and P9); there was, however, a large spread in the Ba and Ma categories.

**Table 8:** The SVR model applied to each individual participant with the independent contribution of each feature group.

			Model R^2^ (%)	Independent contribution of each feature group (%)			
Participant	Musical level	Repetition corr (*r*)	All features	Pitch	Timing	Meter	Phrasing
1	Ma (masters)	–	36.8	7.9	**8.8**	**1.5**	1.4
2	PhD	0.83	**46.8**	**23.2**	2.4	−0.1	**12.1**
3	Ba (bachelors)	0.74	20.7	12.4	0.5	−0.2	6.4
4	Ma	0.43	13.9	6.8	−0.4	0.4	4.0
5	Ma	0.82	37.9	21.3	2.5	1.0	7.1
6	Ba	0.61	33.4	16.4	2.6	−0.4	3.3
7	Ma	0.81	36.7	18.6	1.1	−0.5	7.7
8	PhD (comp)	0.59	20.0	10.6	−0.4	1.1	3.4
9	PhD	0.86	38.8	16.4	2.3	−0.6	9.0
10	Ba	0.34	19.2	10.1	1.2	1.1	4.6
11	Ba	0.35	30.7	18.6	1.7	0.0	3.2
12	Ba	0.84	36.0	18.3	1.8	1.0	11.6
13	Ba	0.77	22.5	11.5	1.2	0.1	7.0
14	Ba	0.74	20.5	11.1	0.5	0.3	4.2
15	Ba	0.75	**40.9**	**29.8**	1.5	−0.1	4.2
16	Ba	0.64	24.4	12.5	**3.7**	−0.1	4.5
17	Ba	0.46	20.9	8.4	0.8	**1.7**	4.2
18	Ba	0.66	27.3	3.7	2.0	0.3	**11.7**
19	Ma	0.64	36.3	18.7	1.5	0.1	6.0
20	Ma	0.40	15.0	7.4	1.7	0.0	2.4

The two highest results in each column in bold. The participants with a resulting *R*^2^ < 20% are greyed out. The third column lists Pearson’s correlations between the first and second recording of melody 1 for each participant.

The similarity between the performances of the first and the second recording of Melody 1 was estimated using Pearson’s correlation (see Table 8). The average correlation was r = 0.65 with a range from 0.34 to 0.86. All correlations were significant (*p* < 0.001). The rather weak correlation for some participants introduces an uncertainty regarding the individual analysis of these participants. It is notable once more that the two professional pianists achieved high correlations, while correlations varied substantially within the other two categories. This suggests the expected influence of experience on the participants’ ability to reproduce consistent performances and to maintain a consistent level of focus during the course of the experiment. Variation in less correlated repetitions may be indicative either of initial nerves, or, conversely, of wearing out of concentration. It may also pertain to technical control, or simply a more spontaneous approach to the task. Despite these inconsistences, however, the Cronbach’s alpha (CA) reported in section 3.5 indicates that there remains a significant agreement between the participants across all melodies.

## Conclusion and discussion

In conclusion, this study shows that performed dynamics can be quantified and predicted to some extent in terms of the different underlying components of the musical structure, that is, in terms of pitch, timing, meter, and phrasing. The experimental method using the same musical material allowed for the formation of an average across the 20 pianists. This was made possible by the sufficiently small differences among the pianists. However, the individual analysis also revealed important differences regarding each participant’s focus on different components. Some participants focused on metrical or rhythmic features, for example, while others focused more on the melodic or phrasal features. This was evident from the data across all melodies and styles. The method of estimating the individual contributions of each component was made possible by the formulation of a large set of features. The same method could be fruitfully applied using other machine learning algorithms, such as XGBoost (Chen and Guestrin, 2016). The component with the largest influence on the dynamics was the pitch-related feature group, concerning pitch differences within small contexts, as well as the broader influence of register. The latter was a dominant factor, often corresponding simply to the *high loud* rule, whereby the higher the pitch the louder it is played. Surprisingly, meter was marked only to a very small degree. Possible explanations for these findings are proposed below.

### Long-term and local variations

A significant challenge in this study was the super positioning of multiple different types of dynamic variations. This meant that both local accents on individual notes, and more long-term variations—such as phrasing and the high-loud principle—were included. All models were optimised by minimising the distance (mean square error) between model and performance. As a result, more long-term variations tended to dominate since they minimise the distance more effectively. This can be observed in Figure 1, where long-term variation is reasonably captured by the model, while note-level variations in some parts are less well captured (see for example the final phrase). It might therefore be useful in the future to design a filter that could separate short and long-term variations so that accent-related variations could be modelled independently.

Another related drawback of the current model is that phrase structure needs to be annotated manually. While considered necessary in this study, such annotation introduces a subjective element to the analysis, making it more complicated to apply consistently to new melodies. It could therefore also be useful to train a model using only the fully automatic features and compare the results.

One possible explanation for why the modelled fit only reached about 65% could be that all melodies (or a large group of melodies) were processed at the same time, while a certain component may only be used in some of the melodies. This was particularly evident for phrasing, with visual inspection indicating that phrase arches appeared in only some of the melodies, even within the same style. Disregarding feature interaction, this means that the model will apply some degree of phrasing to all examples according to the average across the melodies. The *phrasing* rules were also applied with a fixed set of parameters according to a previous experiment, meaning that they were most likely not applied in an optimal way in this data set. Further investigation of different feature sets in relation to the style and period of different pieces would be an interesting path for future studies.

### Influence of meter

The relatively small contribution from *meter* features, as shown in Table 7, was notable. Dynamic accents relating to meter have been identified in performances in several studies (e.g., Lerdahl and Jackendoff, 1983; Drake and Palmer, 1993). One possible explanation for the small influence of meter in this study could be that many other aspects (e.g., rhythmic and melodic grouping) are included in the model. The previously statistically significant influence of meter could thus have been due to an interaction between meter and other inherent features, such as rhythmic and melodic grouping. Lack of accompaniment, whether from the left-hand of the piano part or from another musician or group of musicians—often serving as an explicit marking of the meter—may also have supported this phenomenon. It could equally be due to different strategies used by the participants, as shown in Table 8. For example, in spite of its importance as a structural parameter, a performer may downplay metric emphases in order to focus on the lyrical quality of the music, as opposed to foregrounding its sense of groove or motoric drive.

### Comparison with immanent accents research

The low contribution of meter may also relate to the conscious or unconscious recognition of immanent accents. Meter was shown to be more influential for modelling immanent accents in Friberg et al. (2019) than for the performed dynamics in this study. Friberg et al. (2019) and the present study used the same formulation of the *meter* features, the same modelling method, and partly the same melodies. Still, the influence of meter on the immanent accents was 3.9% (Table 7 in Friberg et al., 2019) and here the influence on the performed dynamics was 0.7% (Table 7). One possible explanation could be that the meter is already emphasised perceptually so that a further explicit emphasis is not necessary (one does not want to emphasise the already obvious).

A similar reasoning can be applied to the last note in each phrase. In the immanent accent study, these notes were marked as very important such that they ‘stick out’ perceptually. Given that there is a strong perceptual impression of these notes, arising from the musical structure, it seems again plausible to assume that it is not necessary to make explicit dynamic accents in such instances. Instead, the last note of the phrase is usually performed softer, aligning with the preceding dynamic phrase curve (e.g., Repp, 1996).

In summary, there appears to be an important interaction between the immanent accents and the performed accents in terms of dynamics, at least with respect to phrasing and meter. A simple and somewhat intuitive principle of performance arising from this could thus be to “not emphasise notes that are inherently salient.” This interaction is, however, likely more complex in reality, when taking into account the different salience levels of the immanent accents.

### Piano technique and participant characteristics

The participants’ feedback shows the significant influence of stylistic, intrinsic, and contextual features of the melodies on their expressive use of dynamics, as identified in our analysis. The responses, as well as subjective identification by the experiment leader of different levels of preparation, and the diverse musical backgrounds and experience of the participants, furthermore highlight the complex interaction of technical, situational, musicological, and cultural factors underscoring each rendition, which may similarly explain the presence of outlying results and the inability of modelling methods, including our own, to ever fully capture the unique qualities of human performance. Nonetheless, the overriding consistency of response to questions of both difficulty and expressive strategy suggests the prevailing reliability of the participant group in informing the conclusions of our analysis.

The generally modest prediction of dynamics among the participants may also be attributed to certain biomechanical limitations and pianistic issues. Some examples, such as Melody 27, did not suggest an obvious fingering pattern. In this instance, quite sophisticated fingering was needed to ensure a consistent performance, requiring both expertise that less experienced participants may not have had (note the first- and third-highest prediction rates for the two professional pianist participants), or preparation time that participants were not willing or able to dedicate to the experiment. To combat this, fingering, or technical performance directions pertaining to other instruments could be added to scores in future experiments to simplify the preparation task and reduce the number of random and outlying variations. Participants could then be advised to follow these directions or alter them where appropriate to suit their technical approach. While it is extremely difficult to guarantee a consistent level of preparation among non-professional participants, as well as to gauge consistency of experience above a certain base level commensurate with the experience of undergraduate first-study pianists during the recruitment process, these adaptations could improve the results of future experiments seeking to generate similarly large datasets in comparable circumstances. A deeper understanding of the relationship between instrumental technique, embodiment, and musical expression, particularly among professional musicians, could also be gained through music-psychological engagement and cross-referencing with a broader range of performer-centric performance studies literature (see for example Doğantan-Dack, 2008).

### Researching other aspects of expressive performance and other instruments

The current study focused solely on dynamics. The same methodology could, however, be productively applied to the study of other aspects of musical performance, such as timing and articulation, in order to gain a fuller picture of the interactive and permeable contribution of each aspect and their relationship to different styles of music. As noted, research in this area has focused principally on the piano but could be usefully expanded by our means to explore the performance of dynamics and other expressive phenomena in different instruments, providing an insight into distinctions between universal and instrumentally contingent aspects of musical performance.

### Participant selection

Finally, the current study highlights the importance of securing as high a level of musical experience in the participants as possible in order to achieve consistent results, and to be able to distinguish clearly between idiosyncratic performance traits and those stemming from a lack of experience or preparation. Nevertheless, gaining data from a high number of participants with a relatively high level of instrumental expertise has been valuable in generating an experimental data set that can be used to identify meaningful trends, as well as unexpected inconsistencies that speak to the unwaveringly personal quality of musical interpretation.

### Data and sound examples

All the data used in this study is available on request. The performances shown in Figure 5 as well the performances of all melodies by some participants, the average of the participants, and the models can be listened to at https://www.speech.kth.se/music/performance/jonesfriberg2023/.

## Data availability statement

The raw data supporting the conclusions of this article will be made available on request, without undue reservation.

## Ethics statement

Ethics approval for the study was granted by the Arts, Humanities, and Communications (AHC) Committee at the University of Leeds. Approved consent forms were signed by all participants for their data and written feedback to be used in publication.

## Author contributions

GJ assembled and formatted the music database and ran the experiment. AF ran the modelling. Both authors wrote the paper.

## Footnotes

^1^ABRSM Grade 8 is a performance qualification commensurate with the expected entry level of first-study musicians on most British undergraduate music programmes.

^2^https://www.speech.kth.se/music/performance/jonesfriberg2023/

^3^Phrase levels 1–3 are reserved for larger musical structures such as sections and movements.
